# Comparison of Job Quality Indices Affecting Work–Life Balance in South Korea According to Employee Gender

**DOI:** 10.3390/ijerph17134819

**Published:** 2020-07-04

**Authors:** Seung-Hye Choi, Eun Young Choi, Haeyoung Lee

**Affiliations:** 1 College of Nursing, Gachon University, Incheon 21936, Korea; hera1004@gachon.ac.kr; 2 Department of Nursing, Graduate School of Chung-Ang University, Seoul 06974, Korea; 11351@naver.com; 3 Red Cross College of Nursing, Chung-Ang University, Seoul 06974, Korea

**Keywords:** job quality, work–life balance, gender, employee

## Abstract

Maintaining a healthy work–life balance is important for both males and females. Nevertheless, gender segregation still exists in labor markets in South Korea. Therefore, this study aimed to investigate differences in occupational characteristics, job quality indices, and work-life balance between male and female employees. This study was a secondary analysis of the data collected through the fifth Korean Working Conditions Survey in South Korea. Generalized ordinal logistic regression analysis was carried out to investigate the associations between job quality indices and work–life balance of employees by gender. The job quality indices were different according to gender. Male employees were mainly affected by working time quality and work intensity, while female employees were affected by both these factors and by the physical environment. Therefore, strategies differentiated by gender are necessary to improve work–life balance. In particular, more careful attention should be paid to female workers’ physical environment.

## 1. Introduction

Healthy work–life balance refers to a state where work activities and home or non-work-related social activities coexist in harmony [1]. The fact that the “work–life balance generation” was selected as one of the top ten keywords that characterize South Korean society in 2018 reflects the values of new generations of office workers who believe overall quality of life is as important as work [2]. 

Maintaining a work–life balance is an important factor for the well-being of both individual workers and their families. Although working hours are decreasing globally, the average annual working time in Korea is 2124 h; among the Organization of Economic Cooperation and Development (OECD) member countries, only Mexico has longer hours, at 2228 h [3]. The average working time in OECD member countries is 1770 h, which is 354 less hours than in Korea [3]. While working hours in South Korea are gradually decreasing, the average working hours of South Korean female workers is about 2000 h, the longest among OECD member countries [3]. Long working hours negatively affect work–life balance and job satisfaction [4]. The OECD has pointed to long working hours as the most important factor that hinders work–life balance in South Korea.

The gender gap in work–life balance and job satisfaction has long been an issue, and various studies have been conducted on it [5,6,7,8,9,10,11]. Although the issue of work–life balance is important for both males and females, females’ economic participation has increased along with social development, while a social expectation that females should be primarily responsible for child-rearing and housekeeping has not changed significantly. According to role congruity theory, traditional values regarding males’ and females’ roles and responsibilities can have an influence on workers’ balance of family and work responsibilities [12]. It has been reported that the role burden of female workers who need to combine work and family life is higher than that of males [13], and that the actual time female workers spend doing housekeeping activities is at least twice that of male workers [14]. 

The labor markets of most welfare developed countries are highly segregated by gender [15,16]. There were differences among the top six occupations for females and males in Europe in 2005 [15]. According to a previous study, extremely female-dominated occupations were office secretaries and nursing professionals, and female-dominated occupations were personal care and related workers, while extremely male-dominated occupations were transport workers and agricultural and other mobile-plant operators, and male-dominated occupations were finance and sales associate professionals [16]. Females’ occupations are predominantly in cities, which might affect the imbalance in sex ratios between city and rural locations [17]. Various studies have been focused on the socioeconomic perspective to use resources at farms [18,19,20,21]; however, work–life balance according to employees’ gender has not been explored yet. In addition to societal role expectations, gender segregation in labor markets also affects the differences in the characteristics of the work environment that affect the work–life balance of male and female workers. The work-related characteristics that affect the work–life balance of married females include job type, the types of workplace, average salary, employment type, average working hours, autonomy within the organization, part-time work, and support from bosses or colleagues [22,23,24,25,26,27]. Self-choice between compensation and flexibility is partly responsible for occupational gender segregation. For example, while males may value a higher salary more than flexibility, the opposite may hold true for females with a working partner present in the household [28].

In cases where work–life balance is not present, individuals may experience a deterioration in their physical and mental health, as well as problems such as decreased quality of life, job satisfaction, and organizational commitment, and companies may experience increased turnover [25,29]. Therefore, it is necessary to survey the factors that affect the work–life balance of males and females separately and plan differentiated measures. This study aims to contribute to the promotion of work–life balance among South Korean employees. Therefore, this study deals with four objectives: (1) to compare the occupational characteristics according to gender, (2) to compare the job quality indices according to gender, (3) to compare the work–life balance according to gender, and (4) to identify the association between job quality indices and work–life balance by gender.

## 2. Materials and Methods 

### 2.1. Study Design

This is a descriptive cross-sectional study to compare job quality indices that affect the work–life balance of employees by gender with secondary data analysis using raw data from the fifth Korean Working Conditions Survey (KWCS) (2017) conducted by the Korea Occupational Safety and Health Agency. 

### 2.2. Study Population

Included in this study were 37,336 (74.4%) employees selected from the 50,205 workers in the fifth KWCS. The selection criteria were: older than 15 years old, and worked for pay for more than one week prior to the time of the survey. Those who were self-employed, business owners, employees, unpaid family workers, and others were considered workers. 

### 2.3. Measures

The variables used in this study were selected by referring to the sixth Euro Working Condition Survey (EWCS) [30]. The survey was composed of five questions about occupational characteristics, 83 questions about six areas of job quality indices, and six questions about work–life balance. 

#### 2.3.1. Occupational Characteristics

Occupational characteristics included age, occupational groups, sectors, number of workers in the workplace, and average monthly income. Sectoral analysis of fifth KWCS was carried out based on the Korean Standard Industrial Classification (KSIC). In this study, the 21 KSIC sectors were reorganized into ten categories based on the sixth EWCS [30].

#### 2.3.2. Job Quality Indices

Job quality indices were divided into six areas: physical environment, work intensity, working time quality, social environment, skills and discretion, and prospects. 

The physical environment included the potential risks to workers’ physical well-being, environmental risks such as vibration and noise, biological and chemical risks such as chemical contact, and ergonomic risks related to posture, which were measured with a total of 15 questions. 

Work intensity included quantitative demands such as the speed of work, time pressure and strict deadlines, factors affecting the performance evaluations, and emotional needs, which were measured with 12 questions. 

Working time quality measured working hours and the length of recovery periods between working days, atypical working hours such as weekend work, night work, and shift work, and the flexibility of working hours in relation to the worker’s discretion to determine working hours and free time to meet business needs. These were measured with a total of 19 questions. 

The social environment measured the positive and negative experiences that can occur in the workplace with 15 questions about experiences of bullying, violence in the workplace, and support from colleagues or superiors.

The area of skills and discretion measured cognitive skills such as task complexity, discretion to decide the order and method of work, and organizational participation and training. These were composed of 15 questions.

Prospects refers to workers’ perception of employment status, type of contract, and prospects for career advancement to measure continuity in the job. A total of seven questions were used to measure prospects. 

The questions were scored with Likert scales or were dichotomous questions. The responses were converted into scores ranging from 0 to 100 points, and the mean index scores were calculated to compare the detailed questions in each area. In this case, the responses were converted into scores so that high scores indicated positive responses. 

#### 2.3.3. Work–Life Balance

Work–life balance was evaluated with a total of six questions. The positive aspect (good fit between working time and non-working time) was measured with the question “Is your working time suitable for you to lead your family life or social life?” using a 4-point Likert scale ranging from “Very appropriate” to “Not appropriate at all”. The negative aspects (the conflicts between work and family) was measured with a total of five statements: “I worry about work even when I’m not working”, “I’m too tired to do housework after work”, “I don’t have enough time to spend with my family members due to my work”, “I don’t have enough time to work because of what happened at home”, and “I feel that I cannot spend time on work because of my responsibility to my family members”. Respondents were asked to rank their agreement with the statements using a 5-point Likert scale ranging from “always” to “not at all”. 

### 2.4. Statistical Analyses

The samples from the fifth KWCS were designed with a secondary probability proportion stratified cluster sample survey, and standardized weights were applied to the samples when the data were analyzed to more accurately estimate the population. The data were analyzed using the IBM SPSS/WIN 25.0 program.

Descriptive statistics and X^2^ tests or t-tests were conducted to identify differences in the occupational characteristics, the job quality indices, and the work–life balance by gender. A generalized ordinal logistic regression analysis, a statistical analysis method for ordinal dependent variables [31], was performed to identify the associations between the job quality indices and work–life balance according to gender. Occupational characteristics were included as control variables. Categorical variables were treated as dummy variables, and monthly average incomes were logit transformed before being applied. 

## 3. Results 

### 3.1. Comparison of Occupational Characteristics According to Gender 

The occupational characteristics of male and female employees are shown Table 1. All the occupational characteristics showed statistically significant differences between the male and female groups. Among age groups, the greatest number of male workers were in their 30s, while the greatest number of female employees were in their 40s. For occupational groups, males were most likely to be “clerks” (24.3%), followed by “professionals” (20.2%) and “plant or machine operators” (16.4%). For females, the greatest number of respondents were “professionals” (27.0%), followed by “clerks” (24.3%) and “service workers” (16.5%). As for sector, the ratio of “industry” employees was the highest for males at 26.3%, and the ratio of “commerce and hospitality” employees was the highest for females at 23.3%. As for number of employees of workplaces, the ratio of male employees in small (1–9 employees) was 31.2% and medium-sized workplaces (10–249 employees) was the highest, exceeding 50%; on the other hand, the ratio of female employees in small-scale workplaces was the highest (48.9%). As for average monthly income, in the case of males, the ratio of those whose monthly income was 3,000,000–3,990,000 won was the highest at 29.7%, while in the case of females, the ratio of those whose monthly income was 1,000,000–1,990,000 won was the highest at 41.3%. 

### 3.2. Comparison of Job Quality Indices According to Gender

All job quality indices showed statistically significant differences between male and female respondents. In the areas of “physical environment”, “work intensity” (for which the score was inverse transformed), and “working time quality”, females showed significantly higher scores than males. In the areas of “social environment”, “skills and discretion”, and “prospects”, males showed significantly higher scores than females. The characteristic of the work environment that showed the largest difference between male and female respondents was “skills and discretion”, for which the score of males was about six points higher than that of females (Table 2). 

### 3.3. Comparison of Work–Life Balance According to Gender

The differences in “work–life balance” between males and females are shown in Table 3. The percentage of female workers who answered that working hours were suitable or very suitable for maintaining a home or social life was 79.4%, which was significantly higher than what was reported by male workers. With regard to the five negative aspect questions, males showed higher ratios than females in the statements “I worry about work even when I’m not working”, “I’m too tired after work to do housework”, and “I don’t have enough time to spend with my family members due to my work”. Females showed significantly higher ratios in the statements “I don’t have enough time to work because of what happened at home”, and “I feel that I cannot spend time on work because of my responsibility to my family members”. 

### 3.4. Associations between Job Quality Indices and Work–Life Balance According to Gender

**Figure 1:**
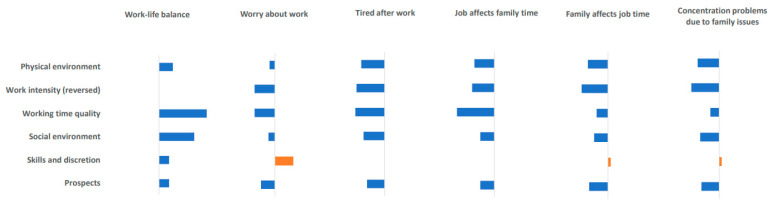
Association between job quality indices and work–life balance (Male).

**Figure 2:**
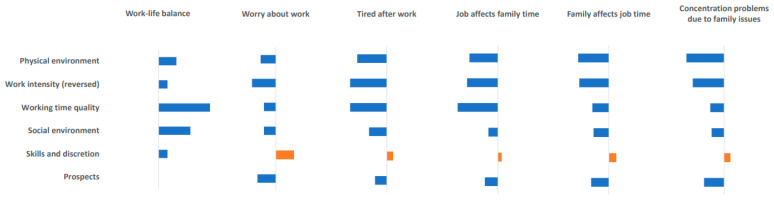
Association between job quality indices and work–life balance (Female).

Table 4 and Figure 1 and Figure 2 show the associations between job quality indices and work–life balance when occupational characteristics were controlled. For the positive item for work–life balance, “working time is suitable for home or social life”, “working time quality” had the largest effect on both males and females (males OR = 1.038, 95% CI = 1.036–1.040; females OR = 1.045, 95% CI = 1.042–1.048). 

Turning to the negative work–life balance items, the “skills and discretion” area was shown to increase worry about work in both males and females. The lower work intensity scores (or higher reverse transformed scores) had the effect of reducing worry about work. For the items “I’m too tired to do housework after work” and “I don’t have enough time to spend with my family members due to my work”, “skills and discretion” had no significant effect on males but had significant positive effects on females. Among the predictors of the effect the job has on time with family members, “working time quality” had the largest effects on both males and females. For the items, “I don’t have enough time to work because of what happened at home” and “I feel that I cannot spend time on work because of my responsibility to my family members”, “skills and discretion” was shown to be a positive predictor in both males and females. Among the predictors of concentration problems due to family issues, the characteristic that had the largest effect was “work intensity” for males, but “physical environment” for females. 

## 4. Discussion

This study was conducted to investigate differences in occupational characteristics, job quality indices, and work–life balance between male and female employees using data from the fifth KWCS. It seeks to understand the associations between job quality indices and work–life balance by gender.

Although the participation of females in the workplace is increasing in South Korea, the occupational characteristics of male and female workers were significantly different in all areas, including age, occupational groups, sectors, number of employees in the workplace, and monthly income. This is consistent with the fact that gender segregation in the labor market is becoming more apparent as females’ paid work increases, as reported in the results of the sixth EWCS [30]. This study found that the commercial and hospital sector are female-dominated, and the industrial sector is male-dominated. These results are similar to those found in a survey of occupational gender segregation conducted in Sweden indicating that extremely female-dominated sectors were nursing and midwifery professionals, and extremely male-dominated sectors were transport workers, building cleaners, etc. [16,32].

Job quality indices also showed significant differences between males and females. Females had higher scores than males in physical environment, work intensity (reversed), and working time quality. Males had significantly higher scores than females in social environment, skills and discretion, and prospects. In the results of this study, the ratios of craft workers and agricultural workers were higher among males, and those of service and sales workers were higher among females. In addition, the ratio of employees in the transport sector was higher among males, but the ratio of employees in health sector was higher among females. Therefore, the difference in job quality indices between males and females may be due to the differences in occupational groups and sectors between males and females.

Overall, finding a good fit between working time and non-working time among the job quality indices was higher among females. When occupational characteristics were controlled, the factor that had the largest effect on a goodness of fit between working time and the non-working time was working time quality for both males and females. The working time quality index takes into account the number of working hours, atypical working time, working time arrangements, and flexibility. According to the results of previous studies, both work–life balance and job satisfaction decreased with longer working hours [4]. Although working hours are decreasing globally, with South Korea seeming to follow this trend, South Korea still has the second-longest working hours among OECD member countries [3]. However, it is also important to consider that improving overall working time quality, including flexibility of working hours, can be more important than simply reducing working hours. This argument is supported by a study conducted in South Korea that after adjusting for confounding variables, a statistically significant association between the working time quality of employees and self-perceived health was found [33]. In addition, it was reported that a reduction in working hours may cause a larger imbalance within the broader economy, but schedule control that allows individuals to select working hours autonomously is effective in achieving work–life balance [34]. 

Most of the job quality indices that positively affected the positive work–life balance items for work–life balance also negatively affected the negative work–life balance items. However, skills and discretion increased both the positive and negative aspects of work–life balance. In particular, skill and discretion were factors that had the most significant positive effect on “worrying about work for work–life balance” for females, although it had no effect on males. It also had a positive effect on the “tired after work” and “job affects family time” items among females. The score for skill and discretion was higher among males than among females. This result, which indicates that skills and discretion have larger effects on work–life balance for females despite the foregoing, was unexpected. In studies conducted outside of South Korea, females reported less decision making authority at work, and their psychological demand level developed more unfavorably than did males”, which resulted in an increased job strain gap between males and females [35]. Previous studies have also reported that in addition to job strain, effort–reward imbalances, such as high efforts and low occupational rewards, are the strongest predictors of poor well-being [36].

The factors of job quality indices that have an effect on work–life balance were investigated by gender. According to the results, males were mainly affected by working time quality and work intensity, while females were greatly affected by the physical environment in addition to these two factors. For females in particular, the physical environment was the factor that had the largest effect on the item “family affects job time”. The physical environment consists of ambient risks, biological and chemical risks, and ergonomic risks. According to a previous study, about 50% of female workers experienced a high prevalence of musculoskeletal symptoms in their lower backs [37]. Although workers’ musculoskeletal symptoms were not directly compared in this study, it was reported that work–life conflicts have a strong positive correlation with musculoskeletal disorders [38]. A previous study found that low physical demand at work is an important factor for a person to remain in work [39]. Therefore, the physical environment needs to be improved to improve the work–life balance of female workers.

This study has several limitations. First, the criteria for selecting survey participants was simply people in South Korea who worked for incomes in the past week and who were at least 15 years of age. This means that the survey included self-employed people, business owners, employees, unpaid family workers, and other workers. Therefore, although standardized weights were applied, the possibility that the results of this study contain biases related to sampling cannot be excluded. Second, although this study investigated differences in occupational characteristics, job quality indices, and work–life balance between male and female employees, differences in job quality indices and work–life balance between male and female employees cannot be simply interpreted as arising from gender differences because occupational characteristics were also different between male and female employees.

## 5. Conclusions

The present situation of jobs and job quality indices is different between male and female employees in South Korea. The factor that had the largest effect on work–life balance was working time quality. Therefore, improving working time quality rather than simply reducing working hours is considered important. In addition, there were differences between males and females in work and family conflict according to job quality indices. Although working time quality and work intensity affected work–life balance for both males and females, females were also affected by the physical environment of their workplace. Therefore, to improve work–life balance, strategies differentiated by gender are necessary. In particular, more careful attention should be paid to the physical environment of female workers. 

## Figures and Tables

**Table 1:** Comparison of occupational characteristics by gender.

Variable	Category	Male		Female		X^2^	*p*
		*n* (%)		*n* (%)			
Age	15–19	233	(1.1)	172	(1.1)	406.402	<0.001
	20–29	3103	(14.6)	3513	(21.7)		
	30–39	5736	(27.1)	3469	(21.5)		
	40–49	5547	(26.2)	3884	(24.0)		
	50–59	4305	(20.3)	3203	(19.8)		
	≥60	2260	(10.7)	1912	(11.8)		
		N = 21,184		N = 16,153			
Occupation	Managers	205	(1.0)	23	(0.1)	4664.509	<0.001
	Professionals	4273	(20.2)	4355	(27.0)		
	Clerks	5143	(24.3)	3927	(24.3)		
	Service workers	1060	(5.0)	2663	(16.5)		
	Sales workers	1608	(7.6)	2216	(13.7)		
	Agricultural workers	121	(0.6)	28	(0.2)		
	Crafts workers	2869	(13.5)	445	(2.8)		
	Plant or machine operators	3482	(16.4)	512	(3.2)		
	Elementary workers	2309	(10.9)	1980	(12.3)		
	Armed forces	113	(0.5)	6	(0.0)		
		N = 21,183		N = 16,155			
Sector	Agriculture	97	(0.5)	56	(0.3)	6566.438	<0.001
	Industry	5578	(26.3)	2114	(13.1)		
	Construction	2667	(12.6)	225	(1.4)		
	Commerce and hospitality	2947	(13.9)	3764	(23.3)		
	Transport	1606	(7.6)	220	(1.4)		
	Financial services	1092	(5.2)	1065	(6.6)		
	Public administration	1265	(6.0)	789	(4.9)		
	Education	1031	(4.9)	2100	(13)		
	Health	588	(2.8)	2946	(18.2)		
	Other services	4318	(20.4)	2874	(17.8)		
		N = 21,183		N = 16,153			
Number of employees	1–9	6557	(31.2)	7834	(48.9)	1408.591	<0.001
	10–249	11,811	(56.2)	7312	(45.6)		
	≥250	2643	(12.6)	880	(5.5)		
		N = 21,011		N = 16,026			
Monthly income	<100	853	(4.1)	2151	(13.4)	7505.939	<0.001
(10,000 won)	100–199	2871	(13.7)	6611	(41.3)		
	200–299	5736	(27.4)	4806	(30.0)		
	300–399	6226	(29.7)	1573	(9.8)		
	400–499	3090	(14.7)	508	(3.2)		
	≥500	2177	(10.4)	369	(2.3)		
		N = 20,953		N = 16,018			

**Table 2:** Comparison of job quality indices by gender.

Indices	Male (*n* = 21,183)	Female (*n* = 16,154)	t	*p*
	M ± SD	M ± SD		
Physical environment	75.20 ± 12.80	79.33 ± 10.21	−35.037	<0.001
Work intensity (reversed)	69.25 ± 14.41	70.60 ± 13.28	−9.415	<0.001
Working time quality	73.09 ± 17.57	78.73 ± 14.45	−34.000	<0.001
Social environment	82.10 ± 7.58	81.38 ± 7.54	9.151	<0.001
Skills and discretion	43.43 ± 18.52	37.96 ± 17.88	28.877	<0.001
Prospects	76.44 ± 14.00	74.57 ± 13.94	12.808	<0.001

**Table 3:** Comparison of work–life balance by gender.

Variables	Male	Female	X^2^	*p*
	*n* (%)	*n* (%)		
**Positive**				
My working hours fit in well or very well with family/social commitments outside work	15,611 (73.8)	12,811 (79.4)	159.039	<0.001
	N = 21,150	N = 16,131		
**Negative ^1^**				
Worry about work when not working	1693 (8.2)	1267 (8.1)	0.027	0.869
	N = 20,679	N = 15,567		
Too tired after work to do household tasks	2370 (11.5)	1671 (10.5)	8.254	0.004
	N = 20,634	N = 15,863		
Job prevents spending time with family	2743 (13.4)	1856 (11.9)	17.743	<0.001
	N = 20,540	N = 15,642		
Family prevents spending time working	1574 (7.6)	1343 (8.6)	10.615	0.001
	N = 20,640	N = 15,682		
Hard to concentrate on job because of my family responsibilities	1654 (8.1)	1370 (8.8)	6.271	0.012
	N = 20,474	N = 15,538		

X^2^: Chi-square test, *p*: *p*-value. ^1^ The proportion represents the share of workers who replied “always” or “most of the time” on a five-point Likert scale that ranged from “always” to “never”.

**Table 4:** Association between job quality indices and work–life balance by gender.

Job Quality Indices	Positive	Negative				
	Good Fit between Working Time and Non-Working Time	Worry about Work	Tired after Work	Job Affects Family Time	Family Affects Job Time	Concentration Problems Due to Family Issues
	OR (95% CI)	OR (95% CI)	OR (95% CI)	OR (95% CI)	OR (95% CI)	OR (95% CI)
**Male**						
Physical environment	1.011 (1.008–1.013)	0.995 (0.993–0.998)	0.980 (0.978–0.983)	0.983 (0.980–0.985)	0.984 (0.982–0.987)	0.983 (0.980–0.985)
Work intensity (reversed)	1.002 (0.999–1.004)	0.981 (0.979–0.983)	0.976 (0.974–0.978)	0.981 (0.979–0.983)	0.979 (0.976–0.981)	0.978 (0.976–0.980)
Working time quality	1.038 (1.036–1.040)	0.989 (0.987–0.990)	0.975 (0.973–0.977)	0.968 (0.966–0.969)	0.991 (0.990–0.993)	0.993 (0.991–0.995)
Social environment	1.028 (1.024–1.032)	0.994 (0.991–0.998)	0.982 (0.978–0.985)	0.988 (0.984–0.992)	0.989 (0.986–0.993)	0.985 (0.981–0.989)
Skills and discretion	1.008 (1.006–1.010)	1.017 (1.015–1.018)	1.001 (1.000–1.003)	1.001 (0.999–1.003)	1.002 (1.001–1.004)	1.002 (1.001–1.004)
Prospects	1.008 (1.006–1.011)	0.986 (0.984–0.989)	0.985 (0.983–0.987)	0.988 (0.985–0.990)	0.985 (0.983–0.987)	0.986 (0.984–0.988)
	N = 20,961	N = 20,499	N = 20,446	N = 20,396	N = 20,477	N = 20,319
**Female**						
Physical environment	1.014 (1.010–1.018)	0.986 (0.983–0.989)	0.975 (0.972–0.978)	0.976 (0.972–0.979)	0.974 (0.971–0.977)	0.970 (0.967–0.974)
Work intensity (reversed)	1.007 (1.004–1.010)	0.978 (0.975–0.980)	0.969 (0.966–0.971)	0.974 (0.971–0.976)	0.975 (0.972–0.977)	0.976 (0.974–0.979)
Working time quality	1.045 (1.042–1.048)	0.989 (0.986–0.991)	0.969 (0.967–0.972)	0.965 (0.963–0.968)	0.986 (0.983–0.988)	0.988 (0.986–0.991)
Social environment	1.025 (1.020–1.030)	0.989 (0.985–0.993)	0.985 (0.981–0.989)	0.992 (0.987–0.996)	0.987 (0.983–0.992)	0.990 (0.986–0.995)
Skills and discretion	1.007 (1.005–1.009)	1.017 (1.015–1.019)	1.005 (1.003–1.007)	1.003 (1.001–1.005)	1.006 (1.004–1.008)	1.005 (1.003–1.007)
Prospects	1.001 (0.998–1.004)	0.983 (0.981–0.986)	0.990 (0.998–0.993)	0.989 (0.986–0.991)	0.985 (0.983–0.988)	0.984 (0.982–0.987)
	N = 15,658	N = 15,129	N = 15,406	N = 15,255	N = 15,262	N = 15,153

OR: Odds Ratio, CI: Confidence Interval.
